# *Plasmodium falciparum* merozoite surface protein 1 block 2 gene polymorphism in field isolates along the slope of mount Cameroon: a cross – sectional study

**DOI:** 10.1186/s12879-015-1066-x

**Published:** 2015-08-05

**Authors:** Tobias O. Apinjoh, Rolland B. Tata, Judith K. Anchang-Kimbi, Hanesh F. Chi, Eleanor M. Fon, Regina N. Mugri, Delphine A. Tangoh, Robert V. Nyingchu, Stephen M. Ghogomu, Theresa Nkuo-Akenji, Eric A. Achidi

**Affiliations:** Department of Biochemistry and Molecular Biology, University of Buea, Buea, Cameroon; Department of Microbiology and Parasitology, University of Buea, Buea, Cameroon; Department of Zoology and Animal Physiology, University of Buea, Buea, Cameroon; Department of Medical Laboratory Science, University of Buea, Buea, Cameroon

## Abstract

**Background:**

Malaria remains a major global health burden despite the intensification of control efforts, due partly to the lack of an effective vaccine. Information on genetic diversity in natural parasite populations constitutes a major impediment to vaccine development efforts and is limited in some endemic settings. The present study characterized diversity by investigating *msp1* block 2 polymorphisms and the relationship between the allele families with ethnodemographic indices and clinical phenotype.

**Method:**

Individuals with asymptomatic parasitaemia (AP) or uncomplicated malaria (UM) were enrolled from rural, semi-rural and semi-urban localities at varying altitudes along the slope of mount Cameroon. *P. falciparum* malaria parasitaemic blood screened by light microscopy was depleted of leucocytes using CF11 cellulose columns and the parasite DNA genotyped by nested PCR.

**Results:**

Length polymorphism was assessed in 151 field isolates revealing 64 (5) and 274 (22) distinct recombinant and major *msp1* allelic fragments (genotypes) respectively. All family specific allelic types (K1, MAD20 and RO33) as well as MR were observed in the different locations, with K1 being most abundant. Eighty seven (60 %) of individuals harbored more than one parasite clone, with a significant proportion (*p* = 0.009) in rural compared to other settings. AP individuals had higher (*p* = 0.007) K1 allele frequencies but lower (*p* = 0.003) mean multiplicity of genotypes per infection (2.00 ± 0.98 vs. 2.56 ± 1.17) compared to UM patients.

**Conclusions:**

These results indicate enormous diversity of *P. falciparum* in the area and suggests that allele specificity and complexity may be relevant for the progression to symptomatic disease.

## Background

Malaria remains a major global human health-threatening disease, resulting in approximately 207 million clinical cases and 627,000 deaths each year, mainly in sub-Saharan Africa [1]. *Plasmodium falciparum*, causes the most severe forms of the disease, is responsible for the high morbidity and mortality, frequent antimalarial drug resistance and aborted vaccines trials [2, 3]. Despite the dramatic decrease in clinical cases from 1,883,199 in 2009 to 313,315 in 2012 [1] malaria still remains a serious public health problem in Cameroon. Although efforts for malaria control and prevention continue to intensity, multiple factors, including insecticide resistance in the mosquito vectors, the emergence and rapid spread of drug-resistant strains and the lack of effective vaccines, are contributing to the global worsening of the malaria situation. Therefore, there is an urgent need for the development of a broadly effective malaria vaccine to reduce malaria morbidity [4] and significantly impact on this disease of enormous public health burden.

Extensive genetic diversity in natural parasite populations is a major obstacle to the development of an effective vaccine against the human malaria parasite, since antigenic diversity limits the efficacy of acquired protective immunity to malaria [5–7]. Individuals born in malaria-holoendemic areas suffer repeated malaria attacks in infancy and it takes 3 to 5 years to develop immunity that confers protection against parasitemia and illness. This may be due to the concomitant infection with different parasite genotypes, bearing numerous allelic forms of asexual blood-stage *P. falciparum* antigens, over successive infections and within a given infection that delay the acquisition of immunity [8]. An infection may thus have multiple different genotypes due to super-infection and mosquitoes inoculating multiple genotypes during a single bite. The extent of multiple-genotype infections sheds light on malaria transmission, parasite diversity, and the development of immunity.

The merozoite surface protein 1 (*msp1*) and other highly diverse single-copy genes have been used to study allelic diversity and estimate the minimum number of different parasite genotypes present within *P. falciparum* infections [9]. The sequence differences and tandem repeat polymorphism result in fine characterization of parasite genotypes [8]. The Block 2 region represents the most polymorphic part of the gene and its sequences may be grouped into one of the three allelic families or variants (*K1*, *MAD20* and *RO33*) [10]. Alleles in *K1* and *MAD20* contain antigenically unique, tripeptide repeats, with extensive diversity in the number of repeats [10]. *RO33* lacks the tripeptide repeats observed in the other two families; however, outside block 2, this allele is similar to the *MAD20* type [11]. Fragment size polymorphism in the three block 2 allele families has commonly been used as a molecular marker in studies of malaria transmission dynamics and host immunity in *P. falciparum* malaria [8, 12–14]. Genetic diversity at the *msp1* locus is further increased due to the high meiotic recombination rates between *MAD20* and *RO33* that creates a fourth allele family known as *MR* [15].

Studies about the malaria parasite and its interaction with the human host are invaluable to effectively combat malaria. Although there have been numerous studies describing the profile of patients and parasite genetics in endemic regions in Africa, South America and Asia [9, 13, 14, 16], there is limited information about the population demographics and parasite genetics in the Mount Cameroon region. Furthermore, recent infrastructural development in the area have led to ecological changes, which together with other factors, such as rainfall, temperature, and humidity, affect the structure of the vector population and thus transmission of infection and probably the genetic diversity of the circulating parasites. Although previous entomologic and parasitologic studies in this region have shown the influence of these changes on the heterogeneity of the malaria transmission pattern, few have determined whether this variability translates into variation in the *P. falciparum* genetic diversity. In addition, studies in the mount Cameroon area have been constrained and yet restricted to the three major *msp1* alleles in children from limited ecological foci [17–19]. Furthermore, there have been no reports on malaria parasite diversity in adults and the contribution of the *MR* allele. This study investigated the distribution of the *msp1* block 2 recombinant and major allele families and their relationship with age, altitude, season and antimalarial use in uncomplicated malaria and asymptomatic parasitaemic individuals along the slope of mount Cameroon. This will enrich the data on parasite population diversity that is invaluable for the design and implementation of an effective malaria vaccine.

## Methods

### Ethics statement

The study was approved by the Institutional Review Board of the Faculty of Health Sciences, University of Buea, Cameroon (No. 2013-03-0153) while administrative authorization was obtained from the South West Regional Delegation of Public Health. Written informed consent or assent was obtained from all participants or the parents/guardians for those below 21 years of age.

### Study area

The study was conducted in localities on the eastern slope of Mt Cameroon, with varying malaria transmission profiles and geographic features (Table 1) [20]. The terrain rises from the Atlantic ocean at the Gulf of Guinea, gradually increasing from Ombe through Mutengene to 800– 1,200 m in Buea. The area is characterised by a forested equatorial climate, modified by the ocean and mountain, comprising two seasons: a short dry season (November–March) and a long rainy season (March-November). Ambient temperatures vary from 18 °C in August to 35 °C in March [19, 21] whilethe relative humidity (75–80 %), average annual rainfall (2625 mm) and precipitation (2,000–10,000 mm) are relatively high [21].

**Table 1:** Geographical characteristics, malaria parasitaemia and clinical profile of the study sites in the Mount Cameroon area

Altitude		Site	Geographic coordinates	Locality	GMPD^a^		
Class	masl^b^				AP	UM	P value
Low	135	Ombe Native	4°06’N, 9°29’E	Rural	9533	39175	0.029
	197	Mutengene	4°08’N, 9°30’E	Semi-Rural			
Interm-ediate	385	Mile 14 - Dibanda	4°11’N, 9°30’E	Semi-Rural	6000	21898	<0.001
	397	Mile 15 - Buea	4°11’N, 9°30’E	Semi-Rural			
	485	Mile 16 - Bolifamba	4°13’N, 9°30’E	Semi-Rural			
	533	Muea	4°17’N, 9°30’E	Semi-Rural			
	575	Molyko	4°15’N, 9°29’E	Semi-Urban			
High	636	Tole	4°11’N, 9°24’E	Rural	5464	17300	0.002
	771	Soppo	4°15’N, 9°25’E	Semi-Urban			
	976	Buea Town	4°16’N, 9°23’E	Semi-Urban			
p value					0.380	0.109	

*AP* Asymptomatic parasitaemia, *UM* Uncomplicated Malaria

^a^ *GMPD* geometric mean parasite density per microliter of blood

^b^ *masl* metres above sea level

Malaria transmission is intense and perennial in the area, with parasitaemia higher in the rainy seasons and at lower altitude [22]. P. falciparum is responsible for most of the malaria infections, with a prevalence of up to 85 % reported recently in asymptomatic adults while P. vivax and P. malaria accounted for 14.9 % and 5.8 % infections respectively [23]. Anopheles gambiae is the dominant, most aggressive and most active of the three malaria vectors (A. gambiae, A. funestus and A. nili) [19, 21], with infection rates and overall Entomological Inoculation Rates (EIR) as high as 287 infective bites/person/year and 3.93 infective bites/person/night respectively [19]. Although the indigenes of this area are of the Bakweri tribe and part of the Bantu ethnic group [24] its fertile volcanic soils and vast plantations have attracted people from other regions of the country, mainly from the Semi-Bantu ethnic group of the North West. There is a substantial level of human migration between localities, mainly for educational, recreational and commercial purposes.

### Selection of sampling sites and participants

**Fig. 1:**
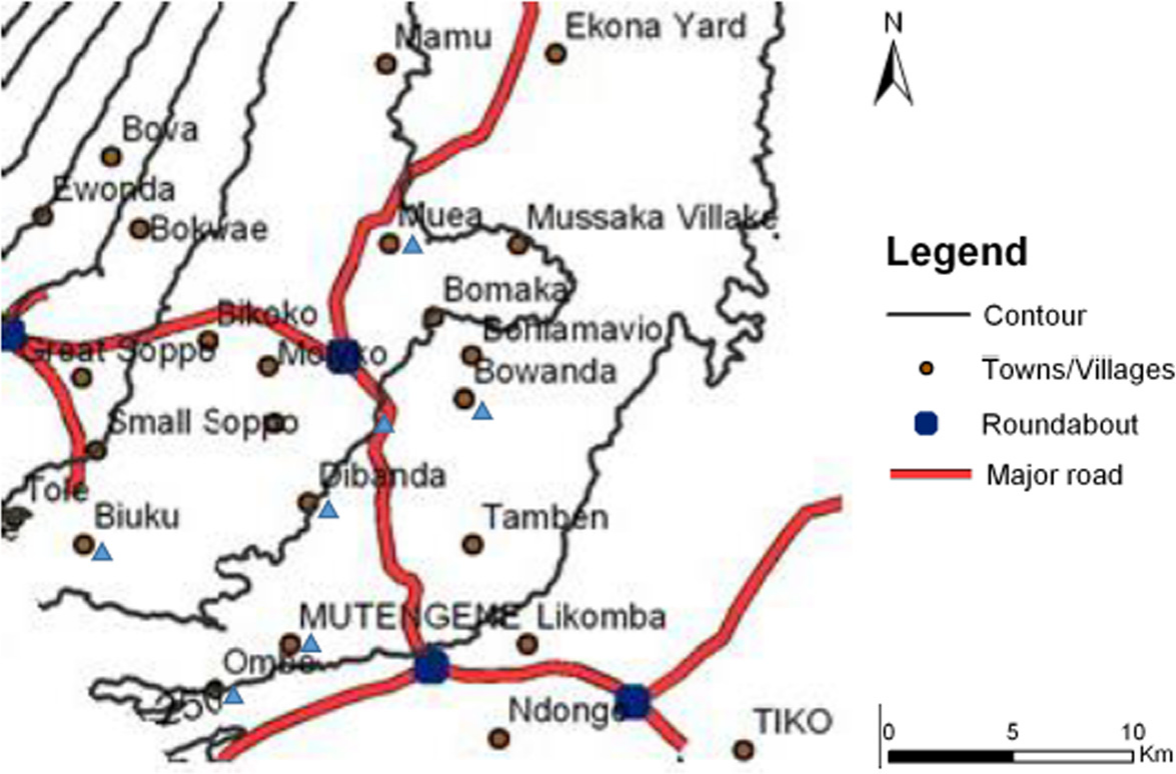
Map of the study area. Localities on the slope of Mt. Cameroon included in the survey are indicated by blue triangles

This cross-sectional community - and hospital - based study was conducted between May 2013 and March 2014. Communities were first identified as rural, semi-rural or semi-urban and then randomly selected based on differences in altitude (Fig. 1). All selected sites were geo-located using a handheld GPS (eTrex, Vista, Garmin, USA); communities below 251 m were considered to be at low altitude while those between 385 – 626 m and above 626 m were at intermediate and high altitude respectively [20]. Individuals with asexual *P. falciparum* infection and no signs/symptoms of the disease, asymptomatic parasitaemia (AP), were enrolled through surveys in randomly selected communities as described elsewhere [20]. Uncomplicated Malaria (UM) subjects were registered from health facilities within these communities andcharacterised by an axillary temperature ≥ 37.5 °C, asexual *P. falciparum* parasitaemia, haemoglobin ≥ 8 g/dL and full consciousness but noclinical signs and symptoms of severe malaria and/or evidence of vital organ dysfunction. A structured questionnaire was used to record demographic and clinical data such as age, area of residence and drug history of all participants.

### Sample collection and parasite detection

Prospective participants were prescreened using finger prick blood samples spotted on glass slides by light microscopy. Thick and thin blood smears were prepared following standard procedures and stained with 10 % Giemsa (Sigma, St. Louis, USA). The malaria parasitaemia status and density were determined under oil immersion with the 100x objective, 10x eyepiece of a binocular Olympus microscope (Olympus Optical Co., Ltd, Japan) while the *Plasmodium* species was identified on thin blood smears. A smear was only considered negative if no malaria parasites were seen in 100 high power fields. With each positive smear, the level of parasitaemia was estimated by counting the parasites against at least 200 leucocytes and assuming a leucocyte count of 8000 per microlitre to calculate the number of parasites/μl blood [22, 25]. Quality control was ensured by staining a known positive and negative sample to ascertain the quality of Giemsa for each freshly prepared stock [25]. Venous blood (3–5 ml) was then collected from P*. falciparum* positive participants into an EDTA tube (BD Vacutainer Systems, Plymouth, UK) for molecular analysis.

### DNA extraction and allelic typing

Leucocytes were depleted from whole blood using CF11 cellulose columns as described by the WorldWide Antimalarial Resistance Network (WWARN) protocol v1.2 [26] with some modifications. Parasite genomic DNA was then extracted using a commercial kit (Qiagen, UK) according to manufacturer’s instructions, eluted with 100 uL TE (10 mM Tris–HCl; 0.5 mM EDTA; pH 9.0) elution buffer (Qiagen, UK) and kept at −34 °C until genotyping. Nested PCR genotyping was performed by amplification of the highly polymorphic Block 2 region of *msp1*, considered to be the most informative genetic marker for the assessment of multiplicity of *P. falciparum* infection [27]. An initial amplification of the outer region of the gene was followed by individual nested PCR reactions using family specific primers for K1, MAD20, RO33 and MR, based on previously described standard protocols [28], with slight modifications. All reactions were carried out in a final volume of 20 μl containing 1X of MgCl_2_ free buffer, 2 mM of MgCl_2_, 125 μM dNTPs, 250 nM of each primer (Table 2) and 0.4 U of Taq polymerase (Sigma, UK). In the first round reaction, 1 μl of genomic DNA was added as a template and the amplification performed in a thermocycler (Biorad T100™, California, USA) viz: initial denaturation at 95 °C for 5 mins, followed immediately by 25 cycles of denaturation at 94 °C for 1 min, annealing at 58 °C for 2 mins and extension at 72 °C for 2 min. The final cycle had a prolonged extension at 72 °C for 5 mins. In the nested reaction, 0.5 μl of the primary PCR product was added as DNA template and the amplification performed similarly to the first round except that the primer concentrations were doubled, the annealing done at 61 °C for 2 mins and the cycles of denaturation – annealing – extension increased to 30. Positive and negative controls were systematically incorporated in each PCR run.

**Table 2:** Sequences of the primers used to amplify the *msp1* gene of *P. falciparum* isolates along the slope of Mount Cameroon

Amplification	Allele	Primer	Primer sequence
Primary PCR			
	NA	Forward	5^I^ - CTAGAAGCTTTAGAAGATGCAGTATTG - 3^I^
		Reverse	3^I^ - CTTAAATAGTATTCTAATTCAAGTGGATCA - 5^I^
Secondary PCR			
	*K1*	Forward	5^I^ - AAATGAAGAAGAAATTACTACAAAAGGTGC - 3^I^
		Reverse	3^I^ - GCTTGCATCAGCTGGAGGGCTTGCACCAGA - 5^I^
	*MAD20*	Forward	5^I^ - AAATGAAGGAACAAGTGGAACAGCTGTTAC - 3^I^
		Reverse	3^I^ - ATCTGAAGGATTTGTACGTCTTGAATTACC - 5^I^
	*RO33*	Forward	5^I^ - TAAAGGATGGAGCAAATACTCAAGTTGTTG - 3^I^
		Reverse	3^I^ - CATCTGAAGGATTTGCAGCACCTGGAGATC - 5^I^
	*MR*	Forward	5^I^ - AAATGAAGGAACAAGTGGAACAGCTGTTAC - 3^I^
		Reverse	3^I^ - CATCTGAAGGATTTGCAGCACCTGGAGATC - 5^I^

*NA* Not Applicable

### Detection of alleles

The secondary PCR products were separated by electrophoresis on 1.5 % agarose gel in 1X TBE (Trisborate EDTA) buffer stained with 0.5 % (v/v) ethidium bromide at 100 V for 20 mins. Bands were visualized under UV transillumination by the gel document system (Gel Doc™, Biorad, California, USA) and fragment sizes estimated by comparison to the 1 kb plus DNA ladder (Invitrogen, UK). The prevalence of each allelic type was determined as the presence of PCR products for the type in the total number of amplified bands. The overall number of genotypes present within the *P. falciparum* population and their respective prevalence was assessed by arbitrarily binning 20 base-pair (bp) intervals together to define a genotype. The median genotype for each allele family was identified and the absolute size of the identified median band +/− 10 bp formed the initial bin. Thereafter, each 20 bp interval below and above the median band were defined as representing a distinct genotype [27].

### Multiplicity of infection and heterozygosity

The multiplicity of infection (MOI), the number of genotypes per infection was estimated as the average number of PCR fragments per individual, by dividing the total number of *msp1* fragments detected by the number of positive samples as described previously [29]. Heterozygosity, the likelihood of being infected by two parasites with different alleles at a given locus was estimated from the following formula: HE = [n/(n-1)] [(1-Σpi^2^)], where n represents the number of samples and pi, the allele frequency at a given locus. Monoclonality was defined as the presence of only one allele of the three major *msp1* types in the sample while isolates with two or more genotype were considered as polyclonal infection [30].

### Statistical analyses

All data were entered into Excel and analyzed using SPSS Statistics 20 for windows (SPSS Inc, Chicago, USA). The significance of difference in prevalence were explored using the Pearson’s *χ*^2^ test whereas the differences in group means were assessed using Student’s t - test or analyses of variance (ANOVA). A difference giving a p value ≤ 0.05 was considered statistically significant.

## Results

### Baseline demographic data

The characteristics of the study participants are presented on Table 3. A total of 259 individuals with microscopy confirmed *P. falciparum* mono-infection were enrolled, mainly with uncomplicated malaria (192, 74.1 %) and asymptomatic parasitaemia (64, 24.7 %). The clinical phenotype of three participants could not be ascertained because their body temperature and/or fever status were not recorded at enrolment. The geographic distribution of the participants included 145 (56 %) mainly from semi-rural areas of mile 16-Bolifamba (59, 22.8 %), mile 14 Dibanda and 15 Buea (28, 10.8 %), Muea (27, 10.4 %) and Mutengene (26, 10 %); 71 (27.4 %) mainly from semi-urban areas of Molyko (41, 15.8 %) and Soppo (20, 7.7 %) and 35 (13.5 %) from rural communities of Tole (26, 10 %) and Ombe (6, 2.3 %). The exact residence of 8 (3.1 %) of the participants within the area could not be ascertained. The mean age (± SD) and geometric mean parasitaemia of the participants was 13.83 ± 13.10 years (range: 4 months - 65 years) and 15,715 parasites/μl (range: 1267–1,840,000) respectively. Most of the study participants were from the semi-bantu ethnic group (72.2 %, 161/223) and had at least 10,000 parasites per microliter of blood (54.9 %, 141/257). One hundred and sixteen (47.2 %) had anaemia while 30.5 % (40/131) had reportedly taken antimalarial drugs.

**Table 3:** Basic characteristics of the study population

Parameter	n	All subjects	n	AP	n	UM	P value
GMPD^a^	256	15715	64	6328	192	21730	0.030
Age [Mean ± SD] (years)	256	13.8 ± 13.1	64	8.7 ± 8.0	192	15.5 ± 14.0	<0.001
Weight [Mean ± SD] (kg)	224	36.6 ± 24.7	61	27.4 ± 18.8	163	40.1 ± 25.9	0.001
Hb [Mean ± SD] (g/dl)	243	10.7 ± 2.1	64	10.1 ± 1.6	179	10.9 ± 2.2	0.006
Anaemia prevalence (%)	116	47.2	38	33.0	77	67.0	0.024
Reported antimalarial use (%)	40	30.5	3	6.5	37	43.5	<0.001
Altitude							
Low	31	12.6	11	35.5	20	64.5	0.118
Intermediate	161	65.2	35	21.7	126	78.3	
High	55	22.3	18	32.7	37	67.3	
Locality							
Rural	34	13.7	19	55.9	15	44.1	<0.001
Semi-rural	144	58.1	39	27.1	105	72.9	
Semi-urban	70	28.2	6	8.6	64	91.4	
Gender							
Female	133	52.4	30	22.6	103	77.4	0.310
Male	121	47.6	34	28.1	87	71.9	
Ethnicity							
Bantu	62	27.8	12	19.4	50	80.6	0.109
Semi-bantu	161	72.2	48	29.8	113	70.2	

^a^ *GMPD* geometric mean parasite density per microliter of blood

*AP* Asymptomatic parasitaemia, *UM* Uncomplicated Malaria

#### *Plasmodium falciparum* allelic diversity

**Fig. 2:**
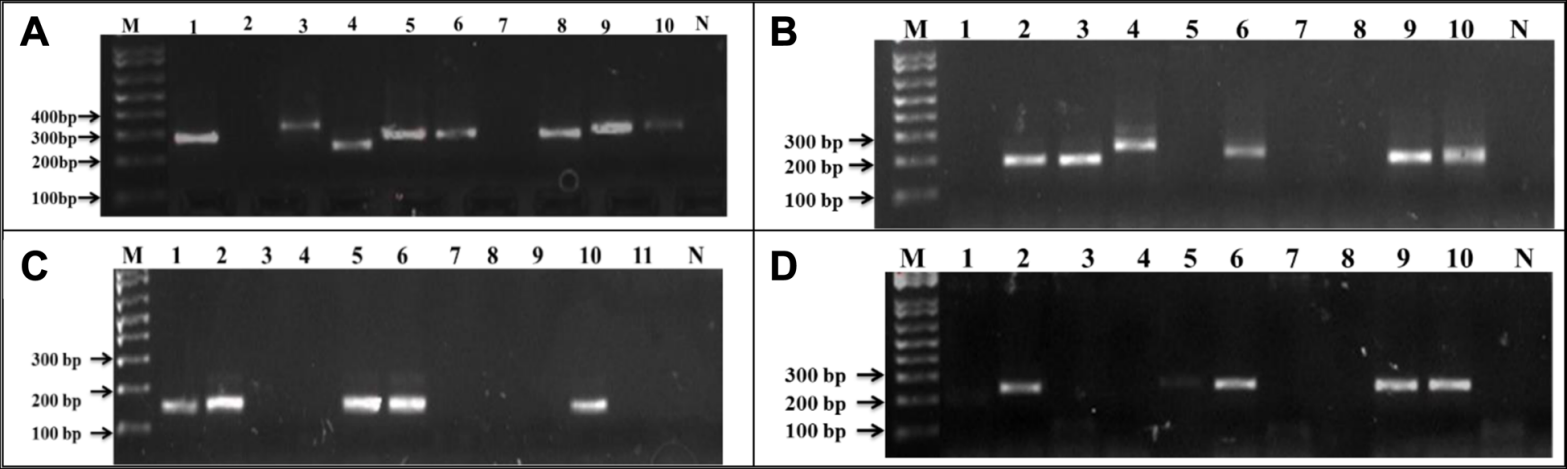
Banding pattern of *msp1* block 2 alleles in asymptomatic and symptomatic *P. falciparum* infections along the slope of mount Cameroon. **A** = *K1*, **B** = *MAD20*; **C** = *RO33;* **D** = *MR;* M = 1 kb plus molecular weight marker; 1 – 11 = selected samples; *N* = negative control

Length polymorphism was assessed in 151 *P. falciparum* field isolates (Fig. 2), with a total of 64 distinct recombinant (*MR*) and 274 major allele family fragments detected respectively, representing an estimated 27 *msp1* genotypes. Six (4.0 %) samples (geometric mean parasitaemia of 6,257 parasites/μl (range: 1660–40,000) were excluded from the analysis due to negative PCR outcome in all allele families.

**Fig. 3:**
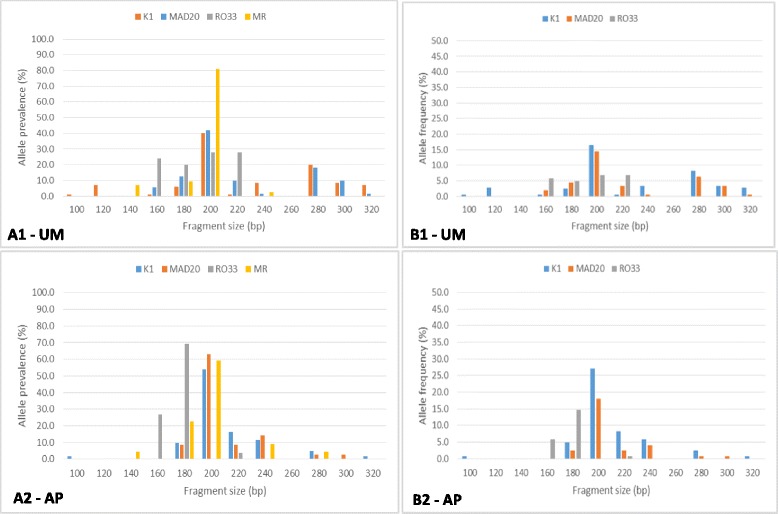
Fragment size polymorphisms (A1 and A2) and allele frequencies (B1 and B2) of *msp1* block 2 allele families in participants with uncomplicated malaria (UM) and asymptomatic parasitaemia (AP) respectively

The distribution of genotypes within the respective allelic families and their corresponding band size ranges in AP and UM individuals is presented on Fig. 3. The *K1* allelic family was the most polymorphic, with thirteen distinct fragments, representing 10 genotypes while *MAD20*, *MR* and *RO33* had 8, 5 and 4 different genotypes respectively. All *msp1* family specific allelic types i.e. *K1*, *MAD20* and *RO33* as well as MR were observed in the different geographical locations (Table 4). In general, the K1 type was most abundant while RO33, the least abundant was detected in 46.2 % of the samples. Eighty seven (60 %) of individuals harbored more than one parasite clone, with some 39.3 % of the infections carrying two allelic types (*K1*/*MAD20*, *K1*/*RO33*, *MAD20*/*RO33*) whereas 30 samples contained all three major *msp1* allelic types*.* The overall prevalence of the *K1*, *MAD20* and *MR* alleles was similar across the different localities whereas the proportion of the *RO33* allele (*p* = 0.007) as well as polyclonality (*p* = 0.009) was significantly different among the localities. In UM patients, all *msp1* allele types were similar across localities while AP individuals in rural settings had the highest proportion of Ro33 (*p* = 0.003), MR (*p* = 0.034) and polyclonal infections (*p* = 0.023) (Table 4).

**Table 4:** Distribution of *msp1* block 2 alleles among study localities

Msp1 block 2 allele type	Clinical phenotype	Number of positives (%)				P value
		All subjects	Rural	Semi-rural	Semi-urban	
K1	AP	55 (90.2)	16 (84.2)	36 (94.7)	3 (75.0)	0.260
MAD20		32 (52.5)	13 (68.4)	18 (47.4)	1 (25.0)	0.170
RO33		26 (42.6)	14 (73.7)	10 (26.3)	2 (50.0)	**0.003**
MR		21 (34.4)	11 (57.1)	9 (23.7)	1 (25.0)	**0.034**
Monoclonality		24 (39.3)	3 (15.8)	18 (47.8)	3 (75.0)	**0.023**
Polyclonality^a^		37 (60.7)	16 (84.2)	20 (52.6)	1 (25.0)	**0.023**
K1 + MAD20		14 (23.0)	3 (15.8)	11 (28.9)	0 (0.0)	0.284
K1 + RO33		6 (9.8)	3 (15.8)	3 (7.9)	0 (0.0)	0.507
MAD20 + RO33		2 (3.3)	2 (10.5)	0 (0.0)	0 (0.0)	0.102
K1 + MAD20 + RO33		15 (24.6)	8 (42.1)	6 (15.8)	1 (25.0)	0.094
K1	UM	58 (71.6)	10 (71.4)	30 (71.4)	18 (72.0)	0.999
MAD20		45 (55.6)	9 (64.3)	24 (57.1)	12 (48.0)	0.591
RO33		38 (46.9)	8 (57.1)	18 (42.9)	12 (48.0)	0.645
MR		38 (46.9)	6 (42.9)	19 (45.2)	13 (52.0)	0.819
Monoclonality		34 (42.0)	3 (21.4)	21 (50.0)	10 (40.0)	0.167
Polyclonality^a^		47 (58.0)	11 (78.6)	21 (50.0)	15 (60.0)	0.167
K1 + MAD20		17 (21.0)	4 (28.6)	7 (16.7)	6 (24.0)	0.578
K1 + RO33		9 (11.1)	2 (14.3)	2 (4.8)	5 (20.0)	0.145
MAD20 + RO33		8 (9.9)	3 (21.4)	3 (7.1)	2 (8.0)	0.279
K1 + MAD20 + RO33		13 (16.0)	2 (14.3)	9 (21.4)	2 (8.0)	0.344

Boldface values indicate significant p values

*AP* Asymptomatic parasitaemia, *UM* Uncomplicated Malaria

^a^Estimated based on the three major allele families only

### Variation in msp1 block 2 alleles with disease phenotype

The proportion of the *K1* allele was higher (*p* = 0.007) in asymptomatic parasitaemia (AP) individuals (55/61, 90.2 %) compared to their uncomplicated malaria (UM) counterparts (58/81, 71.6 %). However, the proportions of *MAD20*, *RO33*, *MR* and polyclonality were independent of disease phenotype. The variation in the proportion of the recombinant and major allele families as well as polyclonality with age, altitude, antimalarial usage, season and level of malaria parasitaemia in UM and AP individuals is shown on Table 5. The frequencies of all the alleles and the proportion of individuals with polyclonal infections was independent of age. Nevertheless, in children below 5 years of age, the frequency of the *MAD20* (*p* = 0.013) and *MR* (*p* = 0.005) alleles was higher in the UM compared to their AP counterparts. Conversely, the proportion of the *K1* allele (*p* = 0.043) was higher in AP children below 5 years compared to their UM counterparts. There was no association between allele proportions with altitude in UM individuals. However, in AP participants, *RO33* (*p* = 0.022) and *MR* (*p* = 0.044) allele frequencies were highest at high altitude (Table 5). At low altitude, the frequency of the MAD20 allele (*p* = 0.009) was higher in UM compared to AP individuals while at intermediate altitude, the latter had higher *K1* (*p* = 0.009) but lower *RO33* (*p* = 0.043) and *MR* (*p* = 0.006) allele frequencies compared to the former. Apart from the higher proportion of the *RO33* allele (*p* = 0.030) in AP individuals during the transition compared to the rainy season, the frequency of alleles was independent of the season of enrolment. During the rainy season, AP individuals had higher *K1* (*p* = 0.017) but lower *MR* (*p* = 0.017) allele frequencies compared to their UM counterparts. In UM patients, the proportion of the *MAD20* allele (*p* = 0.041) as well as polyclonal infections (*p* = 0.041) was higher in non-antimalarial users compared to those who had reportedly taken antimalarial drugs prior to the survey. There was no association between the frequency of alleles or polyclonal infections and the level of malaria parasitaemia (Table 5).

**Table 5:** Variation in *msp1* block 2 diversity with age, altitude, antimalarial usage and season in individuals with Uncomplicated Malaria (UM) and Asymptomatic Parasitaemia (AP); a, b, c, d, e significance of differences in *K1*, *MAD20*, *RO33*, polyclonaility and *MR* proportions in uncomplicated and asymptomatic parasitaemia individuals respectively

Parameter	Uncomplicated Malaria [n (%)]					Asymptomatic parasitaemia [n (%)]					P value				
											a	b	c	d	e
	*K1*	*MAD20*	*RO33*	Polyclonality	*MR*	*K1*	*MAD20*	*RO33*	Polyclonality	*MR*					
Age group (years)															
< 5	11 (65)	14 (82)	9 (53)	12 (71)	11 (65)	16 (94)	7 (41)	7 (41)	10 (59)	3 (18)	**0.043**	**0.013**	0.300	0.473	**0.005**
5 – 9	11 (69)	7 (44)	5 (31)	6 (38)	4 (25)	25 (89)	16 (57)	16 (57)	17 (61)	12 (43)	0.100	0.392	0.447	0.138	0.236
10 -14	8 (73)	4 (36)	4 (36)	4 (36)	5 (46)	7 (100)	6 (86)	6 (86)	6 (86)	4 (57)	0.202	0.057	0.352	0.057	0.500
≥ 15	27 (75)	20 (56)	19 (53)	24 (67)	17 (47)	7 (78)	3 (33)	4 (44)	4 (44)	2 (22)	0.618	0.207	0.470	0.198	0.164
**P value**	0.882	**0.058**	0.423	0.070	0.154	0.450	0.136	0.804	0.414	0.158					
Altitude															
Low	14 (78)	13 (72)	7 (39)	11 (61)	6 (33)	10 (91)	3 (27)	4 (36)	5 (46)	3 (27)	0.356	**0.023**	0.604	0.330	0.534
Intermediate	28 (65)	20 (47)	23 (54)	22 (51)	24 (56)	30 (91)	17 (52)	10 (30)	16 (55)	8 (24)	**0.009**	0.665	**0.043**	0.770	**0.006**
High	16 (80)	12 (60)	8 (40)	14 (70)	8 (40)	15 (88)	12 (71)	12 (71)	14 (82)	10 (59)	0.413	0.501	0.063	0.315	0.254
**P value**	0.383	0.165	0.450	0.354	0.214	0.952	0.080	**0.022**	0.085	**0.044**					
Season															
Transition^a^	11 (85)	8 (62)	5 (39)	8 (62)	4 (31)	16 (94)	11 (65)	11 (65)	13 (77)	9 (53)	0.397	0.579	0.153	0.314	0.225
Rainy	47 (69)	37 (54)	33 (49)	39 (57)	34 (50)	39 (89)	21 (48)	15 (34)	24 (55)	12 (27)	**0.017**	0.489	0.132	0.770	**0.017**
**P value**	0.217	0.636	0.505	0.779	0.203	0.460	0.183	**0.030**	0.116	0.059					
Reported Antimalarial use															
Yes	12 (67)	7 (39)	11 (52)	7 (39)	8 (44)	3 (100)	2 (67)	1 (33)	2 (67)	1 (33)	0.342	0.388	0.684	0.388	0.612
No	15 (71)	15 (71)	7 (39)	15 (71)	9 (43)	38 (91)	25 (60)	21 (50)	28 (67)	19 (45)	**0.059**	0.355	0.859	0.702	0.858
**P value**	0.748	**0.041**	0.399	**0.041**	0.921	0.751	0.651	0.517	0.746	0.585					
Level of parasitaemia (parasites/μl)															
< 10, 000	25 (78)	22 (69)	11 (34)	20 (63)	14 (44)	43 (92)	27 (57)	20 (43)	30 (64)	18 (38)	0.089	0.310	0.465	0.904	0.628
≥ 10, 000	33 (67)	23 (47)	27 (55)	27 (55)	24 (49)	12 (86)	5 (36)	6 (43)	7 (50)	3 (21)	0.157	0.456	0.418	0.736	0.066
**P value**	0.293	**0.053**	0.068	0.510	0.645	0.420	0.153	0.610	0.352	0.201					

Boldface values indicate significant p values

^a^Transition from rainy to dry season

### Association between mean multiplicity of infection, sociodemographic and clinical status

A majority of the infections were polyclonal, with only 58/145 (40 %) of the individuals carrying single *P. falciparum* genotypes (Table 4). The overall mean multiplicity of genotypes per infection (MOI) ± SD was found to be 2.33 ± 1.13 (range: 1–6). Mean MOI was similar (*p* = 0.726) across the different study sites but was higher (*p* = 0.003) in UM patients (2.56 ± 1.17) compared to their AP (2.00 ± 0.98) counterparts. The variation in multiplicity with age, altitude, locality, season, antimalarial usage and level of parasitaemia in UM and AP individuals is presented in Table 6. Uncomplicated malaria patients who had reportedly taken antimalarial (*p* = 0.020) and asymptomatic individuals ≥ 15 years (*p* = 0.039) had lower mean MOI compared to non-antimalarial users and younger counterparts respectively. Additionally, AP individuals < 5 years (*p* = 0.009) and ≥ 15 years old (*p* = 0.007) had lower MOI compared to their UM counterparts. Similarly, MOI was lower in AP individuals at low altitude (*p* = 0.016), in semi-rural settings (*p* = 0.011), during the rainy season (*p* = 0.005) as well as in non-antimalarial users (*p* = 0.023), moderate parasitaemic (*p* = 0.007) and high parasitaemic individuals (*p* = 0.046) compared to their UM counterparts (Table 6). The heterozygosity associated with UM patients and AP individuals was 0.66 and 0.63 respectively.

**Table 6:** Mean Multiplicity of infection across different age groups, altitude, locality, transmission season, reported antimalarial usage and level of parasitaemia in individuals with uncomplicated malaria and asymptomatic parasitaemia

Parameter	Uncomplicated malaria		Asymptomatic parasitaemia		P value
	n	Mean ± SD	n	Mean ± SD	
Age group (years)					
<5	17	3.12 ± 1.54	17	1.82 ± 0.81	**0.009**
5 – 9	16	2.37 ± 1.15	28	2.04 ± 1.04	0.332
10 -14	11	2.18 ± 1.17	7	2.86 ± 1.07^b^	0.224
≥15	36	2.50 ± 0.94	9	1.56 ± 0.73	**0.007**
**P value**		0.142		**0.047**	
Altitude					
Low	18	3.00 ± 1.33	11	1.82 ± 0.87	**0.016**
Intermediate	43	2.33 ± 1.11	33	1.88 ± 1.08	0.082
High	20	2.65 ± 1.09	17	2.35 ± 0.79	0.457
**P value**		0.112		0.218	
Locality					
Rural	14	3.00 ± 1.18	19	2.37 ± 0.76	0.069
Semi-rural	42	2.57 ± 1.35	38	1.87 ± 1.04	**0.011**
Semi-urban	25	2.28 ± 0.74	4	1.50 ± 1.00	0.084
**P value**		0.184		0.111	
Season					
Transition^a^	13	2.85 ± 1.21	17	2.29 ± 0.85	0.193
Rainy	68	2.50 ± 1.17	44	1.89 ± 1.02	**0.005**
**P value**		0.333		0.148	
Reported Antimalarial use					
Yes	18	2.00 ± 0.84	3	2.33 ± 0.85	0.787
No	21	2.86 ± 1.28	42	2.17 ± 1.02	**0.023**
**P value**		**0.020**		0.790	
Level of parasitaemia (parasites/μl)					
<10, 000	32	2.75 ± 1.22	47	2.06 ± 0.99	**0.007**
≥10, 000	49	2.43 ± 1.14	14	1.79 ± 0.98	**0.046**
**P value**		0.230		0.357	

Boldface values indicate significant p values

^a^Transition from rainy to dry season

^b^Significantly higher (*p* = 0.039) than the corresponding values for individuals ≥ 15 years old

## Discussion

The genetic structure of *P. falciparum* populations plays a highly important role in the natural acquisition of immunity in malarial infections. Genetic diversity allows parasites to evade natural immune responses, contributing to the failure of anti-malaria parasite control measures and may jeopardize the effectiveness of vaccines [15]. Therefore, identifying the genotypes circulating in a particular geographical location is necessary to develop strategies to control the disease, including the design of broadly effective vaccines against the parasite [29]. In this study, genetic polymorphisms of the highly polymorphic Block 2 region of msp1 of *P. falciparum* field isolates collected along the slope of mount Cameroon, where malaria is endemic were analyzed. Although the marker has previously been exploited in studying parasites in children from selected foci in the area [17–19] the diversity has been underestimated by only genotyping three allele families as there is a fourth recombinant allele family that is distributed worldwide [7]. This, to our knowledge, is the first report of the contribution of intragenic recombination to *P. falciparum* genetic diversity in wider population spectrum that includes adults.

In addition to all three major msp1 allele families previously reported in the region [17–19], the recombinant between the *MAD20* and *RO33* allele was observed in significant proportion, supporting the notion that sexual intragenic recombination is an important factor in the evolution of genetic diversity [7]. Nevertheless, the *K1* allelic type was found to be the most prevalent, as is the case worldwide. Studies in other areas of the region [17], sub-Saharan Africa countries including neighboring Gabon [31], Nigeria [32] and Congo Brazzaville [33] as well as in Asia as far as Lao PDR [34] and India [35] have consistently shown that the *K1* allele is more prevalent than *MAD20* and *RO33*. Nevertheless, a recent study in the area [19] showed that *K1* was the least abundant of the three major allele types, consistent with previous reports of *MAD20* being the most predominant allele in *P. falciparum* populations from Myanmar, Thailand, Iran, Pakistan and Colombia [29]. Although this low *K1* allele prevalence may have been due to the exclusively asymptomatic infections studied, with their inherently low parasite loads [22], substantial variations in allele prevalence may occur during different study periods, owing to the dynamic nature of the *msp1* genetic structure in *P. falciparum* populations [35]. It is also possible that the acquisition of strain specific immunity may modulate the selection of different allelic variants. Further work is required to consolidate these findings.

Genotype analysis showed a very rich polymorphism of the *P. falciparum* population, with 22 major and 5 MR allelic types (27 genotypes) present at the *msp1* locus, the most common not exceeding 30 % of all alleles of a given family. Nevertheless, this is an underestimation of the diversity in the area since 30.5 % of the individuals reportedly received anti-malarial drugs prior to enrolment and clonal disappearance due to treatment activity cannot be ruled out. Although direct comparison could be difficult due to the differences in the volume of blood samples used in each study, the results are consistent with a fast evolution of mutations in an area of high malaria transmission [35]. Higher diversities of major msp1 alleles (25–33) have been reported in holoendemic areas such as Senegal [36], Uganda [37] and Gabon [31] while in low endemic Asian countries including Thailand [38], Iran [39] and Myanmar [29] parasite diversity is limited to 9–14 genotypes.

The *K1* allelic family was the most polymorphic, with thirteen distinct fragments, representing 10 genotypes while *MAD20* and *RO33* had 8 and 4 different genotypes respectively. These findings are in conformity with previous reports in similar populations in Pakistan [27] where 12, 8 and 5 different *K1*, *MAD20* and *RO33* fragments respectively were observed. Similarly, Bharti *et al.* [35] confirmed the semi-conserved nature of *RO33* in India with only 2 fragments detected while 22 *K1* and 11 *MAD20* were reported. However, in a low transmission area such as Lao PDR, no clonal fluctuation in allelic types are observed in *P. falciparum* clinical isolates, with monomorphic bands observed for all three major *msp1* allele types [34].

The proportion of the *K1* allele was higher in AP compared to UM individuals in this study, consistent with the reported association between the *K1* allele and asymptomatic parasitaemia in children from mile 16-Bolifamba [18] and Nigeria [40]. As such, the *K1* allele type may be responsible for the reduced risk of developing symptomatic disease in AP individuals [40]. Conversely, *MAD20* and *RO33* were found to be associated with UM corroborating the strong association between the *MAD20* allele and symptomatic disease in Ghanaian children [41] as well as the higher proportion of the *RO33* allele in individuals with UM in mile 16-Bolifamba [18]. Although all three major allele families were similar across age groups in both UM and AP, the prevalence of *MAD20* and *RO33* tended to be higher in children below 5 years of age. This suggests that the susceptibility of this vulnerable group of children to UM or symptomatic malaria may accrue to the higher proportion of both allele families. The frequency of the *RO33* allele was also significantly higher at high altitude compared to intermediate and low altitude consistent with previous reports of the allele type being the most prevalent at high altitude [19]. There was a trend of decreasing prevalence of all allele families in UM patients with antimalarial use, consistent with a susceptibility of parasite genotypes to antimalarial treatment. Furthermore, the prevalence of *MAD20* allele and polyclonal infections were significantly lower in individuals who reportedly took antimalarials compared to those who did not. This could be as a result of selection due to drug pressure on parasite population that is clearing this parasite variant.

Although it seems likely that nonreciprocal recombination events, such as replication slippage and gene conversion, during the mitotic (asexual) replication of the parasite also play a plausible role in creating allele variation, allelic diversity of *P. falciparum msp1* is mainly generated by meiotic recombination events involving genetically distinct parasite clones that infect the same mosquito vector. New genotypes and thus increased parasite population diversity do not only accrue to increased number of clones per infected individual but also to the proportion of mixed infections [29]. Multiple clonal infections with different parasite genotypes were identified among isolates in a high proportion (60 %) of participants. This is consistent with extensively polymorphic and mainly multiclonal *P. falciparum* infections elsewhere in the region [19] and in southeast Gabon [31]. As such, individuals in this area are frequently exposed to repeated bites of mosquito vectors transmitting different or multiple parasite clones. The higher proportion of individuals with polyclonal infections in the rural settings, recorded here may reflect greater vector densities accruing to poor sanitation and bushy environments.

The overall mean multiplicity of genotypes per infection (2.33) recorded in this study is comparable to previous findings in the area [19] and to other highly endemic areas in Africa with perennial malaria transmission such as Brazzaville [33] and Tanzania [16]. Although much higher MOI have been reported in areas of high malaria transmission such as Gabon [31] and Mauritania [42] the reported MOI is much higher compared to that obtained in low malaria transmission areas of Lao PDR [34] and Malaysia [30]. Mean MOI was consistently higher in UM patients compared to their AP counterparts. This is in line with previous reports of a positive association between multiplicity and clinical morbidity [3]. UM patients who had reportedly taken antimalarials and asymptomatic individuals aged 15 years and above had lower MOI compared to non-antimalarial users and younger counterparts respectively. This is expected since some parasite genotypes must have been cleared in antimalarial users by the drug, reducing the MOI in this group. Also older individuals are believed to have had substantial previous exposure to some of the parasite clones [3] and so developed antiparasite immunity towards specific parasite genotypes, thus reducing the MOI in individuals aged 15 years and above.

This study has a few limitations. First, it is a cross-sectional study, and inherently there are limitations. Though the sample size is reasonable, mount Cameroon is a populous area and there is a definite possibility of sampling error. Furthermore, the inclusion of participants with a minimum of 1000 parasites per microliter only, may have automatically excluded parasite genotypes inherent in individuals with lower parasitaemia.

## Conclusions

Intragenic recombination contributes to the high polymorphisms in *P. falciparum* populations in the mount Cameroon area. Multiplicity of infection was high, with most individuals harboring more than one parasite genotypes, reflecting both the high endemicity level and malaria transmission in the area. Allele specificity and complexity are relevant for the progression to symptomatic disease. This data is invaluable in understanding the parasite’s population dynamics which could facilitate the development and testing of a broadly effective malaria vaccine.
